# Using Orbital Atherectomy in ST‐Elevation Myocardial Infarction: A Case Report From a Nonsurgical Centre

**DOI:** 10.1155/cric/8852815

**Published:** 2026-03-05

**Authors:** Omar Bajmmal, Prashanth Raju, Naseer Ahmad, Ibrahim Antoun

**Affiliations:** ^1^ Department of Cardiology, Kettering General Hospital, Kettering, UK, nhs.uk; ^2^ Department of Life Sciences, University of Leicester, Leicester, UK, le.ac.uk

**Keywords:** acute coronary syndrome, nonsurgical centre, orbital atherectomy, STEMI

## Abstract

**Background:**

Coronary artery calcification significantly complicates percutaneous coronary intervention (PCI), particularly in acute myocardial infarction (AMI), where rapid revascularisation is essential. In ST‐elevation myocardial infarction (STEMI) with heavily calcified culprit lesions, conventional strategies often fail due to lesion rigidity. Orbital atherectomy (OA) has emerged as an effective technique for modifying calcified plaques and improving stent delivery. However, its use in STEMI and in nontertiary centres such as district general hospitals (DGHs) remains underreported.

**Case Presentation:**

A 61‐year‐old man with a history of smoking and hypertension presented with inferior STEMI and hemodynamic instability. Coronary angiography revealed heavy calcification and occlusion of the proximal right coronary artery (RCA). Initial attempts using standard wires, microcatheters, and parallel wiring were unsuccessful. The lesion was successfully crossed using a ViperWire, enabling OA. Subsequent adjunctive therapies achieved optimal vessel preparation and revascularisation, including intravascular lithotripsy (IVL), intravascular ultrasound (IVUS)–guided stenting, and postdilation. The patient recovered uneventfully and was discharged after 2 days, remaining asymptomatic and compliant with secondary prevention at 6 weeks. This case highlights the feasibility of OA in STEMI with severe calcification in a DGH without onsite surgical cover. Careful planning, operator expertise and multidisciplinary collaboration enabled a favourable outcome despite procedural challenges.

**Discussion:**

The case stresses the need for broader access to advanced PCI tools, enhanced training and standardised protocols in resource‐limited settings. Further studies and real‐world data are essential to evaluate the long‐term safety and efficacy of OA in acute settings.

## 1. Background

Coronary artery calcification poses a significant challenge in percutaneous coronary intervention (PCI), particularly in the context of acute myocardial infarction (AMI), where timely restoration of flow is critical. Among AMI presentations, ST‐elevation myocardial infarction (STEMI) with heavily calcified lesions in culprit vessels requires advanced strategies to achieve successful revascularisation. Conventional methods, such as balloon angioplasty and stenting, often fall short in such scenarios due to lesion rigidity and resistance to dilation.

Orbital atherectomy (OA), a device‐based method of plaque modification, has emerged as a potential solution for addressing calcified lesions [1], enabling better vessel preparation and stent delivery [2]. Although its use is well documented in elective PCI, reports of its application in the acute STEMI setting are scarce, largely due to the complexities and risks inherent in an emergent setting. OA trials are primarily conducted in tertiary care centres, and little evidence is available on their use in acute settings in district general hospitals (DGHs) for STEMI [3, 4].

This case report highlights the successful use of OA in a DGH without an onsite surgical cover for a patient presenting with inferior STEMI and severe calcification of the right coronary artery (RCA). The report underscores the procedural challenges encountered, the decision‐making process and the eventual favourable clinical outcome. This contributes to the growing evidence for OA as a viable option in managing calcified coronary lesions during STEMI.

## 2. Case Presentation

**Figure 1:**
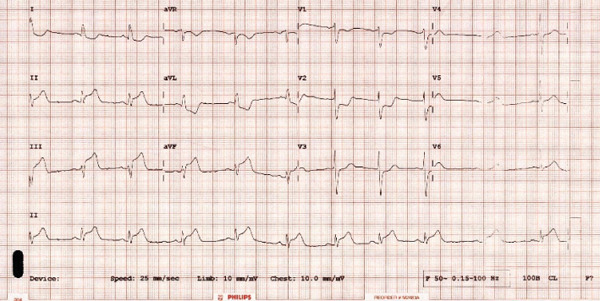
12‐lead electrocardiogram on presentation.

**Figure 2:**
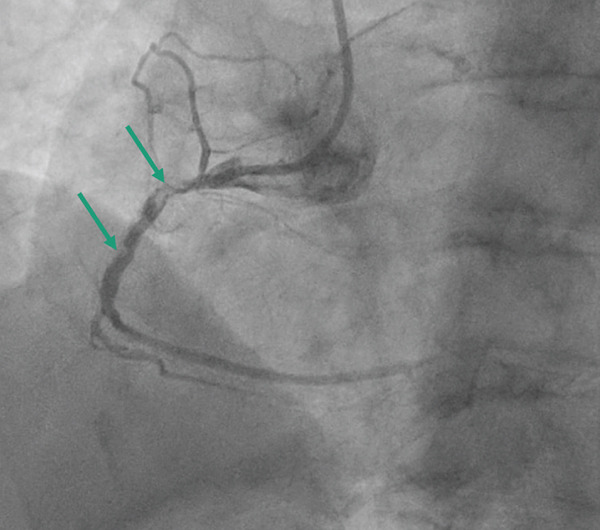
Baseline coronary angiography of the right coronary artery in the right anterior oblique projection demonstrating proximal vessel occlusion with severe calcification, indicated by arrows. Image are acquired prior to wire crossing during primary PCI.

**Figure 3:**
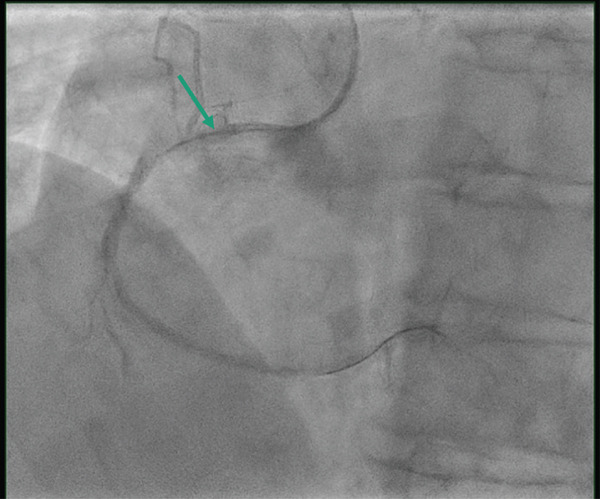
Fluoroscopic image during primary PCI showing orbital atherectomy being performed in the proximal right coronary artery. The position of the atherectomy crown during plaque modification is indicated by arrows. Image are acquired after successful lesion crossing with the ViperWire and before balloon dilatation.

**Figure 4:**
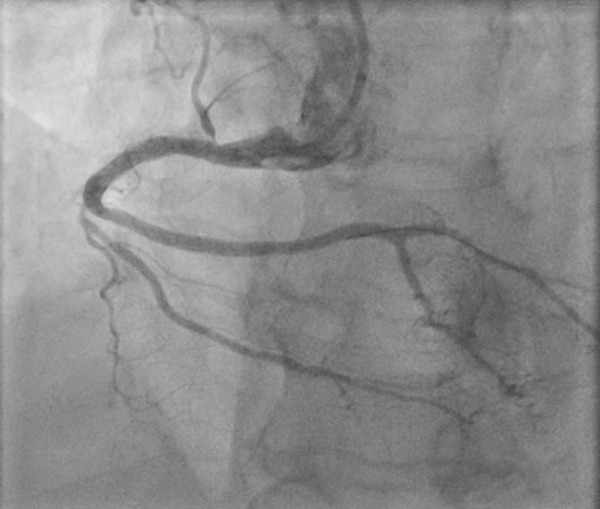
Final angiographic result of the right coronary artery in the right anterior oblique projection following IVUS‐guided stent implantation and high‐pressure postdilation. There is TIMI 3 flow with no residual stenosis. Image are acquired at completion of the procedure.

Our patient is 61 years old with a background of smoking and hypertension. Presented with acute‐onset chest pain with an electrocardiogram (ECG) demonstrating inferior ST elevation and hemodynamic instability (Figure 1). On presentation, the patient was haemodynamically unstable, with systolic blood pressure in the low 80 s mmHg and clinical features consistent with cardiogenic shock complicating inferior STEMI. There was no requirement for cardiopulmonary resuscitation. Initial management included cautious intravenous fluid resuscitation and low‐dose vasoactive support to maintain perfusion during coronary intervention. Serum lactate was mildly elevated, consistent with hypoperfusion and improved following restoration of coronary flow. Temporary pacing was considered, given the inferior infarct territory, but was not required, as there was no high‐grade atrioventricular block or sustained bradyarrhythmia. The patient received aspirin and a P2Y12 inhibitor loading dose prior to coronary angiography. Procedural anticoagulation was achieved with unfractionated heparin, targeting an *a* *c* *t* *i* *v* *a* *t* *e* *d* *c* *l* *o* *t* *t* *i* *n* *g* *t* *i* *m* *e* > 250 *s*, and was monitored throughout the procedure. Coronary angiogram showed left main stem (LMS) normal and moderate mid‐left anterior descending (LAD) disease, predominantly calcific occlusive lesion with a low angiographic thrombus burden in the proximal RCA (Figure 2). We used a 6‐Fr sheath through right radial access, AL 0.75 6Fr guide. We used a workhorse wire (Sion) but were unable to cross the lesion. We then managed to cross with a fielder. We were unable to cross any balloon using 1.0‐mm Sapharie. Routine aspiration thrombectomy was therefore not performed, and glycoprotein IIb/IIIa inhibition was avoided because of the absence of large visible thrombus and the increased bleeding risk in the acute setting. The decision to proceed with OA was made after failure of conventional wiring and balloon‐based strategies, with the aim of modifying severe calcification to enable device delivery and timely reperfusion. OA was performed using the Diamondback 360 system with a 1.25‐mm crown (Figure 3). Low‐speed ablation was used initially, with short runs of approximately 15–20 s, and a total of three passes were performed under continuous haemodynamic and angiographic monitoring. A standard flush solution containing heparin, nitroglycerin and verapamil was used to minimise vasospasm and distal embolisation. Following atherectomy, intravascular lithotripsy was performed using 2.5 mm and 3.0‐mm IVL balloons, delivering a total of 80 pulses to further modify deep calcium prior to stent implantation. Intravascular ultrasound (IVUS) demonstrated severe concentric calcification of the proximal RCA, with a calcium arc exceeding 270° and significant calcium thickness limiting initial balloon expansion. Reference vessel diameters measured approximately 4.0 mm proximally and 3.5 mm distally, guiding stent sizing. Following IVUS‐guided stent implantation and high‐pressure postdilation, the minimal stent area was satisfactory with good circumferential expansion and no evidence of edge dissection or significant malapposition. Final angiography demonstrated TIMI 3 flow with no residual stenosis (Figure 4). There was prompt resolution of ST‐segment elevation on postprocedure electrocardiography. Peak cardiac biomarkers were consistent with an inferior myocardial infarction. Transthoracic echocardiography prior to discharge showed preserved left ventricular systolic function with an estimated ejection fraction of approximately 50% and mild inferior wall hypokinesia. No in‐hospital complications occurred, including no perforation, slow or absent reflow, ventricular arrhythmia or access‐site complications. The estimated symptom‐to‐wire‐crossing time was approximately 180 min, with a door‐to‐balloon time of 48 min, consistent with contemporary STEMI reperfusion targets. The patient was then admitted to the coronary care unit (CCU), where he stayed for 2 days and was then discharged without notable complications. Mechanical circulatory support was also considered; however, it was not pursued because rapid haemodynamic improvement occurred once flow was restored in the RCA. A follow‐up 6 weeks later was reassuring; the patient was asymptomatic and compliant with secondary‐prevention medications. The patient remains under routine cardiology follow‐up, with planned longer‐term clinical surveillance through outpatient review and secondary prevention monitoring to assess for recurrent symptoms, stent‐related complications, and adherence to guideline‐directed medical therapy.

## 3. Discussion and Conclusion

Coronary artery calcification presents significant challenges during PCI, especially in AMI. The rigidity of calcified plaques often hinders balloon angioplasty and stent delivery, complicating revascularisation and increasing the risk of procedural failure [5]. These challenges are magnified in patients presenting with STEMI, where timely restoration of coronary flow is critical to minimising myocardial damage and improving outcomes [6].

OA in the STEMI setting carries specific procedural risks that warrant careful consideration. Distal embolisation of calcific or thrombotic debris may precipitate slow or no reflow, particularly in the prothrombotic milieu of AMI. Coronary perforation is another recognised risk due to aggressive plaque modification in rigid, eccentric or tortuous vessels. In this case, these risks were mitigated through deliberate technique and adjunctive strategies. Short, low‐speed atherectomy runs were performed with continuous haemodynamic and angiographic monitoring to limit distal debris burden. Flow was reassessed after each pass, enabling early detection of slow flow and avoiding prolonged ablation. Adjunctive noncompliant balloon dilatation and intravascular lithotripsy were used to achieve further calcium modification at lower mechanical stress. IVUS‐guided stent sizing and optimisation reduced the risk of under‐expansion and vessel injury. Careful case selection, operator experience and readiness to manage complications were central to the safe use of OA in this acute setting [3]. Although well documented in elective settings, its use during STEMI remains less well established, largely due to concerns about procedural complexity, hemodynamic stability and the risks associated with using OA in emergent settings. These concerns are further amplified in DGHs that may lack the infrastructure of tertiary care centres, including onsite surgical cover [1]. However, a recent study in Portugal demonstrated promising results in a nonsurgical centre [4].

The choice of OA over alternative calcium modification strategies was based on anatomical and procedural considerations. Rotational atherectomy was considered but was deemed less suitable due to significant vessel tortuosity, difficulty advancing microcatheters and the need for wire exchange in a haemodynamically unstable STEMI setting. OA enabled plaque modification with a single dedicated wire, bidirectional sanding of eccentric calcium and incremental lumen gain using short, controlled runs. This facilitated lesion preparation while minimising prolonged manipulation within an acutely occluded vessel. It should be acknowledged that there are currently no randomised trials or guideline recommendations supporting the routine use of atherectomy in AMI. Available evidence is limited to observational studies, case series and real‐world experience. As such, the use of OA in this context should be viewed as a selective, bail‐out strategy in highly selected cases rather than standard practice, underscoring the need for further prospective data.

This case demonstrates the successful use of OA in a DGH setting without onsite surgical backup for a patient presenting with inferior STEMI and severe calcification of the RCA. The procedural challenges included an inability to cross the calcified lesion with standard guidewires and microcatheters. Advanced techniques, including the use of a ViperWire and OA, eventually enabled successful plaque modification and revascularisation. The case was further supported by adjunctive therapies, including IVL and IVUS‐guided stenting, which ensured optimal stent expansion and improved overall outcomes [7, 8].

The absence of onsite surgical coverage necessitated meticulous planning and precise execution, underscoring the importance of operator expertise and teamwork in managing complex cases. The favourable outcome in this case underscores the feasibility and safety of advanced techniques, such as OA, in DGHs, provided that adequate procedural support, clinical judgement and multidisciplinary collaboration are in place. This case contributes to the growing body of evidence supporting the use of OA in STEMI with severe calcification, even in resource‐limited settings. It underscores the need for broader access to advanced PCI tools, enhanced operator training and the development of protocols to standardise the use of OA in such scenarios. Further studies and registries that capture real‐world experiences with OA in acute settings are needed to evaluate its safety, efficacy and long‐term outcomes across diverse clinical settings.

Several plaque modification strategies can be considered for heavily calcified culprit lesions in STEMI, each with distinct advantages and limitations. Noncompliant, scoring or cutting balloons are often first‐line, but they may fail to cross or expand in rigid, concentric calcium and they can increase the risk of dissection when forced. Intravascular lithotripsy can be effective for deep calcium fractures, has a favourable safety profile, and is increasingly used in acute coronary syndromes; however, it requires balloon delivery across the lesion and is therefore less useful when initial crossing is not possible. Rotational atherectomy is well established for severe calcification and can create an initial lumen in uncrossable lesions; however, in STEMI, it raises concerns about distal embolisation, slow or no reflow and procedural complexity, particularly in tortuous vessels and when wire exchange is required. OA offers an alternative that can provide incremental lumen gain and modify eccentric calcium using short, controlled runs over a dedicated wire, which may be advantageous when device delivery is challenging. Overall, the comparative evidence in acute MI remains limited and largely observational; therefore, the optimal strategy is case‐specific and depends on lesion morphology, deliverability, haemodynamic stability and operator experience. In this case, OA enabled initial lesion modification and device delivery, whereas IVL and IVUS then supported safer optimisation of stent expansion.

This case report highlights the successful use of OA in the management of a STEMI patient with severe coronary calcification in a nontertiary centre without onsite surgical cover. Despite the procedural complexity and inherent risks associated with the use of OA in an acute setting, meticulous planning, advanced technical skills and multidisciplinary collaboration facilitated a favourable outcome. The case underscores the feasibility of employing OA in STEMI patients with heavily calcified lesions, even in DGHs, provided that appropriate expertise and procedural support are available. The findings add to the growing evidence supporting the role of OA in acute coronary syndromes, particularly in resource‐limited settings. However, further studies and real‐world data are essential to establish standardised protocols, assess long‐term safety and efficacy and expand access to advanced PCI techniques in nonsurgical centres. Use of artificial intelligence is increasing in cardiology [9, 10] and may further support complex PCI by improving lesion assessment, calcium quantification, interpretation of intravascular imaging and procedural decision‐making. In the future, AI‐driven tools could help identify patients who may benefit from advanced plaque modification strategies and assist operators in real time to optimise safety and outcomes in acute and nonsurgical settings.

**Nomenclature**
PCI: primary coronary intervention
OA: orbital atherectomy.
LAD: left anterior descending
IVL: intravascular lithotripsy
RCA: right coronary artery
DGH: district general hospital
NC: noncompliant
AMI: acute myocardial infarction

## Author Contributions

Ibrahim Antoun: conceptualization, data curation and writing—original draft. Omar Bajmmal: writing—review and editing. Prashanth Raju: writing—review and editing. Naseer Ahmad: writing—review and editing. Omar Bajmmal, Prashanth Raju and Ibrahim Antoun: critical in conceptualising this paper.

## Funding

No funding was received for this manuscript.

## Disclosure

Omar Bajmmal, Prashanth Raju and Ibrahim Antoun are responsible for the first draft. Omar Bajmmal, Prashanth Raju and Naseer Ahmad are responsible for the patient′s clinical and procedural management and conceptualising this manuscript.

## Ethics Statement

The patient gave written informed consent to publish this report in accordance with the journal′s patient consent policy.

## Consent

The authors confirm that written consent was obtained before submission of the case report. The patient gave written informed consent for their clinical details along with any identifying images to be published in this study.

## Conflicts of Interest

The authors declare no conflicts of interest.

## Data Availability

The data that support the findings of this study are available from the corresponding author upon reasonable request.
